# Genome-wide association studies for feed efficiency, production and feeding behavior traits in Canadian purebred Duroc pigs

**DOI:** 10.1093/jas/skag148

**Published:** 2026-05-09

**Authors:** Belle Kim, Duy Ngoc Do, Mohsen Jafarikia, Dan Tulpan, Deborah Adewole, Ghader Manafiazar, Brian Sullivan, Justin Holl, Younes Miar

**Affiliations:** Department of Animal Science and Aquaculture, Dalhousie University, Truro, NS B2N 5E3, Canada; Department of Animal Science and Aquaculture, Dalhousie University, Truro, NS B2N 5E3, Canada; Department of Animal Science and Aquaculture, Dalhousie University, Truro, NS B2N 5E3, Canada; Canadian Centre for Swine Improvement, Ottawa, ON K1V 0M7, Canada; Department of Animal Biosciences, University of Guelph, Guelph, ON N1G 2W1, Canada; Department of Animal Biosciences, University of Guelph, Guelph, ON N1G 2W1, Canada; Animal and Poultry Science, University of Saskatchewan, Saskatoon, SK S7N 5A8, Canada; Department of Animal Science and Aquaculture, Dalhousie University, Truro, NS B2N 5E3, Canada; Canadian Centre for Swine Improvement, Ottawa, ON K1V 0M7, Canada; Pig Improvement Company, Hendersonville, TN 37075, United States; Department of Animal Science and Aquaculture, Dalhousie University, Truro, NS B2N 5E3, Canada

**Keywords:** pig, genome-wide association studies, feed efficiency, production, feeding behavior, single nucleotide polymorphisms

## Abstract

This study aimed to identify potential genetic variants and candidate genes associated with feed efficiency (FE), production, and feeding behavior traits in Canadian purebred Duroc pigs. Genome-wide association studies (GWAS) were conducted using 8,861 individuals and an imputed Affymetrix PigGen Canada 50K panel v2.0 using a linear mixed model (LMM) and a Bayesian B model. This analysis used an adjusted *P*-value threshold (ranging from 6.6 × 10^−5^ to 1.3 × 10^−4^) using a false-discovery rate to determine significance. The number of significant SNPs identified for each trait was as follows: average daily gain (ADG, 48), daily feed intake (DFI, 85), feed conversion ratio (FCR, 101), residual feed intake (RFI, 37), residual gain (RG, 64), residual intake and gain (RIG, 55), backfat thickness (BF, 100), loin depth (LD, 6), Kleiber’s ratio (KR, 0), total time spent eating per day (TPD, 7), and number of visits to the feeder per day (NVD, 6). Several traits (BF, DFI, FCR, RFI, RG, and RIG) showed strong overlapping signals on chromosomes 7 and 10 with 24 shared significant SNPs, indicating potential shared genetic mechanisms. These traits also had 71 overlapping candidate genes, such as *PACSIN1, PTCH1, ADIPOR1*, and *ITPR3*, associated with glucose, lipid, and cholesterol metabolism. Well-known candidate genes in literature associated with growth and fatness such as *MC4R* and *CDH20* were also identified to be associated with ADG, BF, FCR, and DFI in this study. Gene ontology enrichment analysis revealed that a set of the candidate genes were involved in the gonadotropin-releasing hormone (GnRH) and the platelet-derived growth factor (PDGF) signaling pathways. Overall, this study contributed to understanding the genetic architecture and provided a biological foundation for improving FE, production, and feeding behavior traits in Canadian Duroc pigs, facilitating the selection of more efficient pigs.

## Teaser

The findings from this study contribute to a better understanding of the genetic and biological mechanisms behind feed efficiency, production, and feeding behavior traits in pigs that may be applied to improve pig breeding programs in the future.

## Graphical Abstract

**Graphical Abstract:**
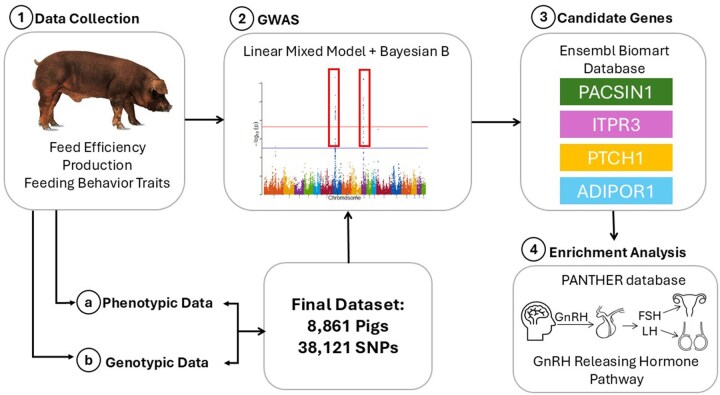

## Introduction

The pork industry is extremely important for Canada’s economy and for ensuring the nation’s food security. In 2024, Canada produced 2.34 million tonnes of meat and exported 6.77 million hogs, resulting in a total profit of $785.9 million (Government of Canada 2025). To support the growing demand for pork in the next few decades, advancements in breeding strategies to improve the quality and quantity of selected pigs are required. In the last 50 years, rapid advancements in pig breeding have been made to meet these growing demands, evolving from selection based on visual appraisal to identifying genetic markers through genome-wide association studies (GWAS) and to applications of genomic selection (Mote and Rothschild 2020).

Feed efficiency (FE) traits and production traits such as average daily gain (ADG), backfat thickness (BF), and loin depth (LD) are highly valued due to their direct economic impact and therefore highly targeted during selection. FE is commonly defined as the ability of an animal to convert feed consumed to weight gain but can also represent the efficiency in using dietary nutrients for maintenance, lean gain, and lipid accretion (Patience et al. 2015). Since feed-related costs account for approximately 60%–70% of the total cost of production, selecting feed-efficient pigs can reduce these costs by optimizing feed utilization (Patience et al. 2015), ultimately resulting in overall improved production efficiency. FE can be assessed in a variety of ways, but in pigs it is most commonly evaluated using the feed conversion ratio (FCR) due to its ease of calculation and interpretation. FCR is defined as body weight gain per unit of feed consumed (Soleimani and Gilbert 2020). Residual feed intake (RFI) is an alternative measure of FE representing the difference between the actual and the expected feed intake required for the animal’s growth and maintenance (Koch et al. 1963). Feeding behavior traits such as the number of visits to the feeder per day (NVD) and the total time spent eating per day (TPD) are important factors that affect FE (Ding et al. 2018). Due to their low to moderate phenotypic and genotypic correlations with FE traits, it is beneficial to include both FE and feeding behavior traits for GWAS to identify shared molecular and genetic factors and ultimately improve genomic selection for these traits (Do et al. 2013).

Currently, numerous studies have used GWAS to identify SNPs and candidate genes associated with various economically important traits in pigs. Notably, many significant SNPs associated with FE and feeding behavior were previously found on *Sus scrofa* chromosomes (SSC) 1, 7, and 12 (Ding et al. 2018; Silva et al. 2019). Among many candidate genes located on these chromosomes, the *MC4R* gene on SSC 1 is well known for its function in regulating ADG and fat accumulation as well as growth and feed intake traits (Kim et al. 2000; Davoli et al. 2012; Silva et al. 2019). The identification of candidate genes with clear and direct functional relevance to traits of interest, such as *MC4R* remains rare, whereas various FE, production, and feeding behavior traits have yet to yield similar results. Therefore, research in the identification of strong candidate genes for these economically important traits is highly valuable, as it can lead to the enhancement of genomic prediction and improved selection accuracy (Shi et al. 2022). For example, GWAS can identify important genomic regions that may be given more weight in genomic prediction models, thus improving their accuracy (Strandén and Jenko 2024). Likewise, there are numerous biological pathways associated with these traits, including hormonal and digestive gland secretion during feeding, olfactory transduction, adipose and muscle tissue development, and lipid metabolism (Do et al. 2014; Ding et al. 2018; Zhuang et al. 2019). However, these economically important traits are rarely explored simultaneously in the same populations, and GWAS studies for lesser-known FE metrics such as residual gain (RG), residual intake and gain (RIG), and Kleiber’s ratio (KR) remain largely unexplored, leading to a gap in knowledge in the identification of associated genetic variants of such traits. Previously, Do et al. (2026) calculated moderate heritabilities for FE traits and moderate to high for production and feeding behavior traits. Generally, FE traits showed weak genetic correlations with feeding behavior traits and varying degrees of strength from low to moderate correlations with production traits, depending on the combination. This project aimed to build off the previous study by identifying single nucleotide polymorphisms (SNPs) and candidate genes associated with FE, production, and feeding behavior traits in Canadian Duroc pigs using univariate GWAS to better understand the biological mechanisms underlying these traits.

## Materials and methods

Historic phenotypic and genotypic data from 2015 to 2022 were obtained from Olymel’s Alphagene farms located in Quebec, Canada, which were acquired by Pig Improvement Company (PIC) in 2022. As all data were collected as part of routine commercial operations and the animals were cared for under the guidelines recommended by the code of practice of the Canadian Council on Animal Care (2009); no further review was necessary by the Animal Care and Use Committee.

### Animals and phenotypic data

Resource population, phenotypic data, and animal models for the estimation of breeding values were described in detail in our previous study (Do et al. 2026). In brief, 15,423 pigs having both feeding records and performance measures were used as based data, which were collected over 7 years was provided by PIC (Quebec). Pigs were fed the same diet *ad libitum*, and once they reached approximately 120 kg, they were either sent to commercial production or a processing facility, unless selected for breeding. Using an automatic feeding system (FIRE® feeders, Osborne Industries Inc., or IVOG® feeders, Hokofarm group), the DFI, TPD, and NVD were measured over on average 80 days. The pig’s body weight was measured individually at the beginning and at the end of this testing period. Only pigs with a minimum of 60 consecutive days of feed intake data were included in the dataset. The remaining eight traits (ADG, BF, LD, RFI, FCR, KR, RG, and RIG) were calculated based on the available data as outlined by Do et al. (2026) using R version 4.3.1 (R Core Team 2023). Before any analysis, the dataset was thoroughly examined to ensure its quality. Animals with missing records for any of the studied traits as well as phenotypic outliers that are 3.5 standard deviations above or below the mean were omitted from the final dataset to ensure the removal of true outliers. Pedigree records were available for 116,221 animals, including 1,134 sires and 5,634 dams spanning over 24 generations. A single-trait animal model with herd-year-season, sex, initial body weight and age as fixed effects and pen, maternal, common litter, additive genetics, and residuals as random effects in ASReml-R version 4 were used to calculate the estimated breeding values (EBVs; Butler et al. 2023; Do et al. 2026). The following formula was used to calculate the reliability of the EBVs:


$$\mathrm{Reliability}=1-\frac{\mathrm{PEV}}{\sigma_{a}^{2}},$$

where PEV represents the prediction error variance, while $\sigma_{a}^{2}$ is the additive genetic variance. Observations with reliability of less than 0.1 were omitted as it indicated a limited pedigree impacting the deregression of the EBVs. The deregressed EBVs (dEBVs) were derived by following the methods described by Garrick et al. (2009) and used as pseudo-phenotype inputs for the GWAS models. After initial quality control, phenotypic data from 14,939 individuals were available for GWAS. From this dataset, only those with EBVs calculated from their own records were retained, resulting in traits with 8,860 to 8,861 observations. Table 1 presents the descriptive statistics of the traits’ dEBVs and their reliability.

**Table 1:** Descriptive statistics of dEBVs for feed efficiency (RFI, FCR, DFI, RG, RIG, and KR), production (ADG, BF, and LD), and feeding behavior traits (TPD and NVD) in Canadian Duroc pigs.

Traits	Abbreviation	N	Mean	SD	SE	CV	Min	Max	Mean r^2^ +SD
**Residual feed intake**	RFI	8,861	−0.055	0.415	0.004	−7.599	−2.831	2.828	0.13 ± 0.074
**Feed conversion ratio**	FCR	8,861	−0.100	0.366	3.888 E-03	−3.657	−2.358	4.072	0.17 ± 0.072
**Average daily gain**	ADG	8,861	0.003	0.084	8.922 E-04	27.976	−0.638	0.842	0.18 ± 0.053
**Daily feed intake**	DFI	8,861	−0.103	0.441	4.682 E-03	−4.263	−8.183	5.223	0.17 ± 0.069
**Backfat thickness**	BF	8,861	−1.566	3.604	0.038	−2.301	−12.347	22.113	0.27 ± 0.065
**Loin depth**	LD	8,861	0.667	15.354	0.163	23.009	−116.017	116.456	0.07 ± 0.068
**Kleiber’s ratio**	KR	8,861	5.000 E-06	0.004	3.76E-05	641.202	−0.1308	0.0403	0.12 ± 0.061
**Residual gain**	RG	8,861	0.013	0.089	9.471E-04	7.008	−1.569	1.573	0.14 ± 0.063
**Residual intake and gain**	RIG	8,861	0.453	2.893	0.031	6.389	−18.873	22.052	0.13 ± 0.073
**Total time spent eating per day**	TPD	8,861	0.460	20.821	0.221	45.220	−79.443	117.768	0.36 ± 0.058
**Number of visits to the feeder per day**	NVD	8,860	−0.022	10.570	0.113	−489.905	−203.067	246.894	0.16 ± 0.070

*Note*. *N*, number of animals; SD, standard deviation; SE, standard error; CV, coefficient of variation; Min, minimum; Max, maximum, *r*^2^, reliability.

### DNA isolation, genotyping, quality control

Ear tissue samples from 16,395 individuals were sent for genotyping to Rapid Genomic (Gainesville, USA), Genome Quebec (Montréal, Canada), Eurofins (Toronto, Canada), DNA Landmarks (Montréal, Canada), and the University of Guelph (Guelph, Canada). Rapid Genomics used low-density SNP panels with 3,500 or 1,200 markers selected from the Affymetrix 50K panel (Affymetrix, Santa Clara, CA, United States). Genome Quebec and Eurofins also used the Affymetrix 50K panel (Affymetrix, Santa Clara, CA, United States), while DNA Landmarks and the University of Guelph used the Illumina 60K panel (Illumina Inc., San Diego, CA, United States). All genotypes were imputed to the Affymetrix PigGen Canada 50K panel v2.0 based on *Sscrofa* 11.1 (Warr et al. 2020) SNP positioning using the FImpute 3.0 software (Sargolzaei et al. 2014). SNPs with a minor allele frequency below 0.01, individuals with a call rate under 0.9, and SNPs failing the Hardy-Weinberg test (*P* < 0.0001) were removed using PLINK 1.9 (Chang et al. 2015) to ensure quality control. The remaining 38,121 SNPs were retained for further analysis. Therefore, the final dataset used for GWAS comprised 8,860 (for NVD) and 8,861 pigs (for the remaining 10 traits) with38,121 SNPs.

### GWAS analysis model

Associations between individual SNPs and dEBVs were assessed using a linear mixed model (LMM) implemented in the Genome-wide Complex Trait Analysis (GCTA) software (Yang et al. 2014). Although the genomic relationship matrix accounts for population structure and environmental variation was accounted for during dEBV calculation, extra caution was considered to reduce the risk of inflation by applying principal components analysis (PCA) using the GCTA software (Price et al. 2006). The first ten principal components were set as covariates to serve as an additional conservative measure against false-positive associations. The LMM model was defined as:


y = a + bx + g + e,

where **y** is the vector of dEBV for each trait, ***a*** is the mean, ***b*** is the fixed effect of the candidate SNP to be tested for association, ***x*** is the SNP genotype indicator variable coded as 2, 1, or 0 for genotypes AA, AB and BB, **g** is the polygenic effect and ***e*** is the residual. It was assumed that ***g*** and ***e*** follow normal distributions$\left(g∼N(0,G\sigma_{g}^{2})\right)$ and $\left(e∼N(0,I\sigma_{e}^{2})\right)$ in which $\sigma_{g}^{2}$ represents the additive polygenic variance and $\sigma_{e}^{2}$ is the residual variance. ***G*** denotes the genomic-based relationship matrix, and ***I*** is the identity matrix. The false discovery rate (FDR) was used to determine the *P*-value threshold, as the Bonferroni correction method tends to set an overly stringent threshold for multiple testing (Johnson et al. 2010). The following FDR formula was used to calculate the significance threshold of the *P*-value for each trait:


$$P=\mathrm{FDR}\times \;\frac{n}{m}$$

where ***FDR*** was set to 0.01, ***n*** represents the number of SNPs with *P* < 0.01 resulting from GWAS, and ***m*** is the total number of SNPs (38,121).

GWAS was also conducted using Bayesian models, specifically BayesB available in the R package hibayes (Yin et al. 2025) using the same dataset. The BayesB approach assumes that only a small proportion of SNPs have non-zero effects on the trait, with their variances following an inverse chi-square distribution (Meuwissen et al. 2001). It is commonly used for FE traits in pigs due to its consideration of different genetic variances for different markers, allowing for accurate reflection of the biological situation of quantitative traits (Meuwissen et al. 2001; Onteru et al. 2013; Zhang et al. 2018; Jung et al. 2024). Non-overlapping genomic windows of 500 kb were used, with a total of 50,000 Markov chain iterations and a burn-in period of 10,000 iterations. The significance threshold for the windows was set to windows posterior probability of association (WPPA)>0.95. The results from both methods were then visualized by Manhattan plots generated with the R packages fastman (Paria et al. 2022) and CMplot (Yin et al. 2021). The percent phenotypic variance explained by each SNP was calculated only for SNPs identified by both the LMM and BayesB models using the following formula:


$$\mathrm{Var}.\;\;\text{Exp}=(\frac{2pq(b^{2})}{V_{p}})\times 100$$

where ***p*** represents the allele frequency of the major allele, ***q*** represents the allele frequency of the minor allele, ***b*** is the SNP effect, and $V_{p}$ is the phenotypic variance.

### Candidate gene annotation, gene ontology and pathway enrichment analysis

Genes overlapping 500 kbp downstream and upstream from the region where the significant SNPs were located were annotated using the Ensembl Biomart database from the R package biomaRT based on *Sscrofa* 11.1 reference genome assembly (Durinck et al. 2009). Gene Ontology (GO) enrichment analysis was performed using the PANTHER classification system (Thomas et al. 2003), based on the list of named candidate genes identified for each trait. Therefore, resulting in the identification of biological pathways and their contributing candidate genes.

## Results

### GWAS with LMM

The dEBVs for all traits in the final dataset are summarized in Table 1. Animals that are missing genotypic information or with dEBVs derived only from the pedigree were removed before GWAS analysis. Quantile-quantile (Q-Q) plots were used to assess the influence of population structure during GWAS analysis. Systemic bias can be identified by checking for deviations of observed -log_10_  *P*-values resulting from the LMM from the expected -log_10_  *P*-values. Most plots did not indicate the presence of systemic bias, showing an expected inflection at the right tail. However, LD, TPD, and NVD showed deviations starting from the midpoint (Figure S1), leading to the inspection of lambda values. The lambda values (genomic inflation factor) were close to 0.9 and the absence of multiple clusters in the population stratification analysis (Figure S2) suggests no evidence of systematic bias in these traits as well.

**Figure 1:**
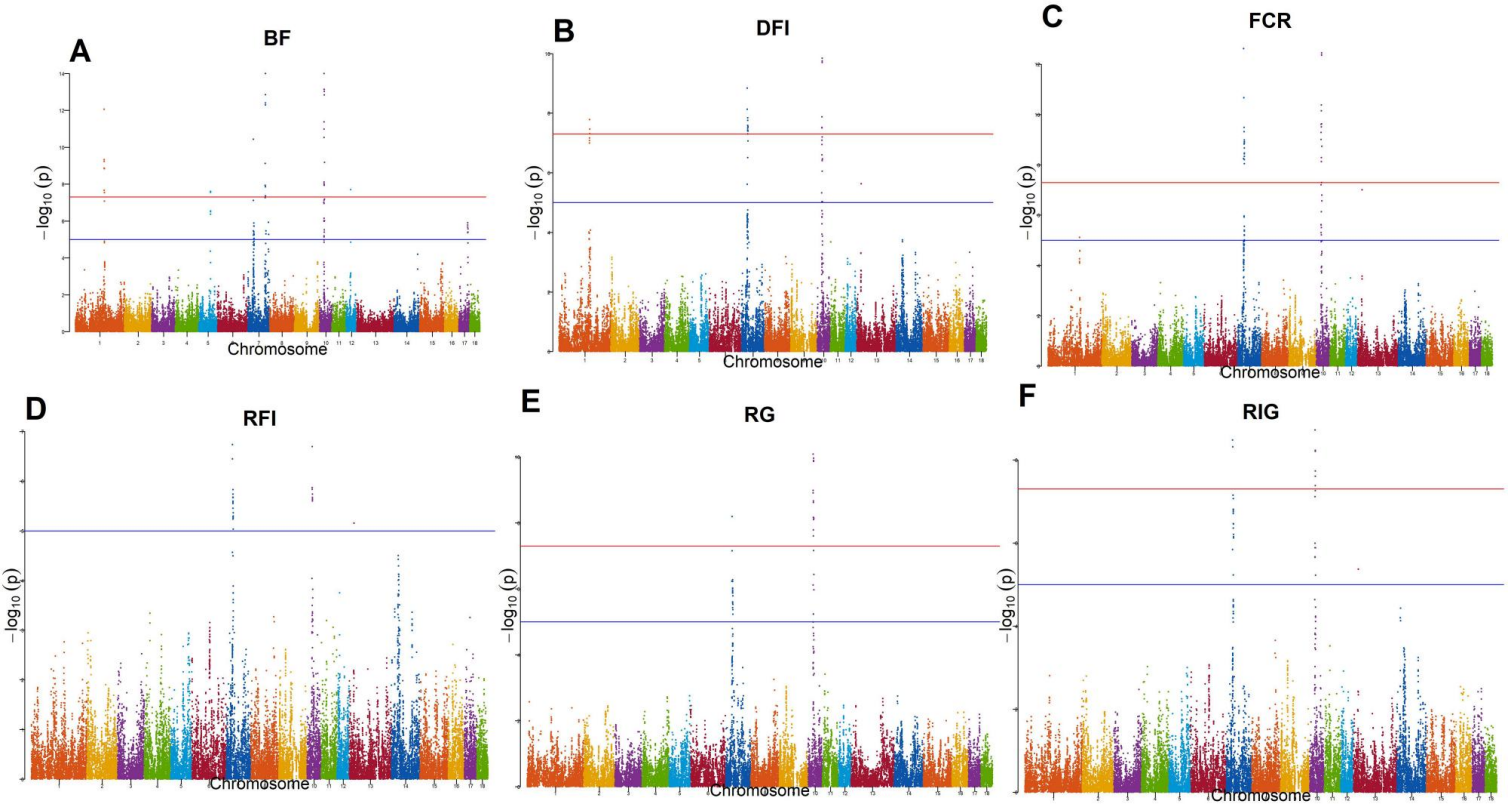
Manhattan plots of (A) Backfat thickness; (B) Daily feed intake; (C) Feed conversion ratio; (D) Residual feed intake; (E) Residual gain; (F) Residual intake and gain (RIG) obtained using Linear Mixed Model (LMM).

GWAS was run with 10 principal components included in the model as covariates, and the results are summarized in Table 2. The resulting Manhattan plots of BF, DFI, FCR, RFI, RG, and RIG are depicted in Figure 1 while the remaining traits are depicted in Figure S3. The empirical *P*-values from multiple testing for ADG, DFI, BF, LD, FCR, RFI, KR, RG, RIG, TPD, and NVD were 9.55e-5, 1.33e-4, 1.14e-4, 1.10e-4, 1.19e-4, 1.20e-4, 6.58e-5, 9.00e-5, 1.13e-4, 9.57e-5, and 9.44e-5, respectively. Overall, 24 shared significant SNPs between BF, DFI, FCR, RFI, RG, and RIG were found on *Sus scrofa* chromosomes (SSCs) 7 at 30.3–30.9 Mb, 33.1–33.8 Mb, and 34.4–34.5 Mb, and on SSC 10 at 24.9 Mb and 26.0–26.7 Mb, respectively. Genes within the 30.0–31.0 Mb of SSC 7 and 26.0–27.0 Mb of SSC 10 were further visualized based on Ensembl *Sscrofa* 11.1 shown in Figures S4 and S5.

**Table 2:** Number of significant SNPs, distribution across chromosomes, and most significant SNPs for each trait obtained from linear mixed model (LMM).

Traits	Significant SNPs	SSC	Most significant SNP
**RFI **	37	7,10,13,14	7:30993927
**FCR**	101	1,7,10,13	7:30502880
**ADG **	48	1	1:161853405
**DFI **	85	1,7,10,13	10:26699078
**BF **	100	1,5,7,10,12,14,17	7:97618073
**LD **	6	5,7	7:97584287
**KR **	0	–	−
**RG**	64	7,10	10:24927504
**RIG**	55	7,10,13,14	10:26091506
**TPD **	7	1,6,14	6:30303413
**NVD **	6	2,3,4	4:105312765

*Note*. RFI, residual feed intake; FCR, feed conversion ratio; ADG, average daily gain; DFI, daily feed intake; BF, backfat thickness; LD, loin depth; KR, Kleiber’s ratio; RG, residual gain; RIG, residual intake and gain; TPD, total time spent eating per day; NVD, number of visits to the feeder per day; SSC, Sus scrofa chromosome. SNPs under the column most significant SNP is denoted as chromosome: basepair.

### GWAS with BayesB

**Figure 2:**
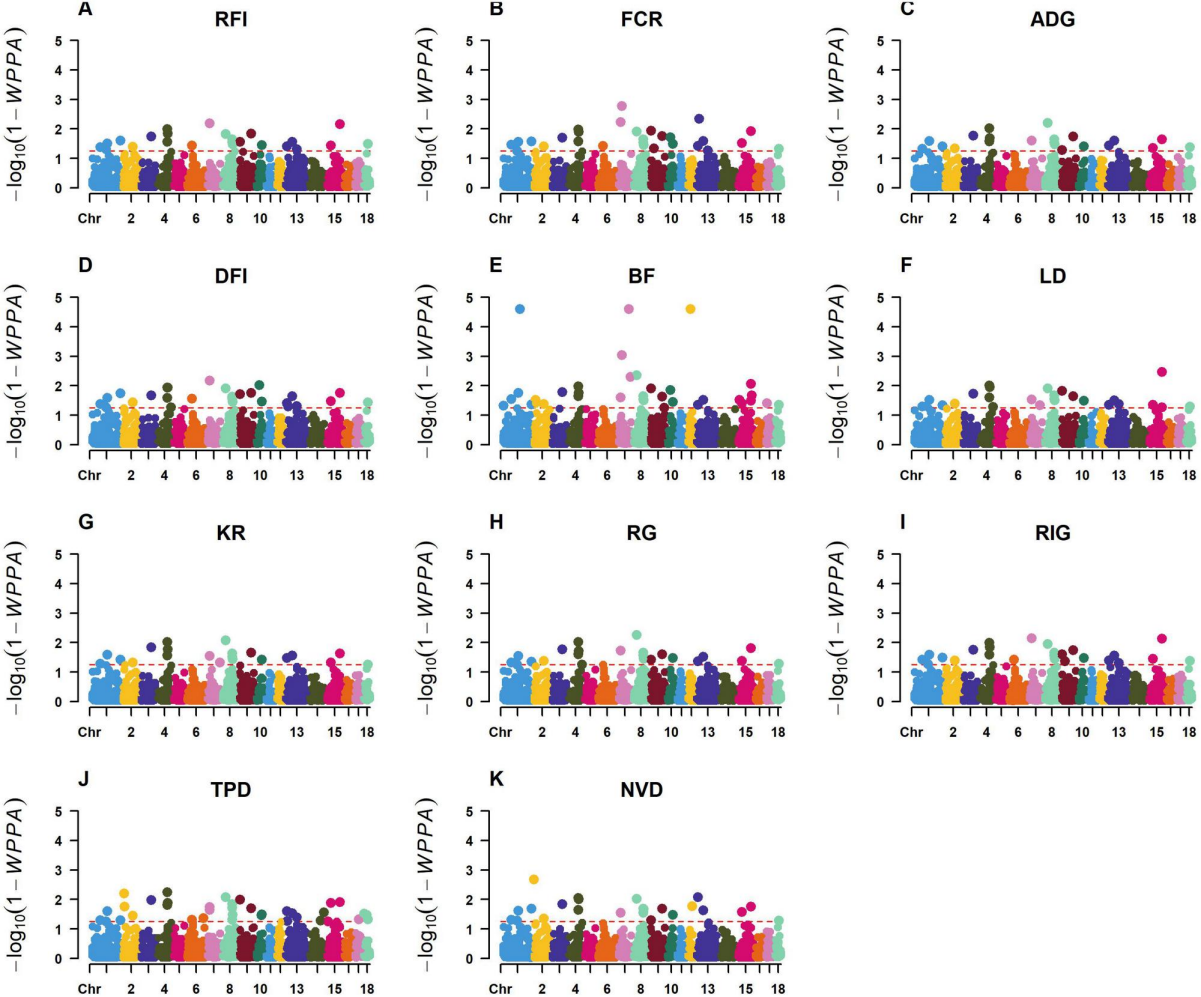
Manhattan plots from GWAS via Bayes B for (A) Residual feed intake; (B) Feed conversion ratio; (C) Average daily gain; (D) Daily feed intake; (E) Backfat thickness; (F) Loin depth; (G) Kleiber’s Ratio; (H) Residual Gain; (I) Residual intake and gain; (J) Total time spent eating per day; (K) Number of visits to the feeder per day.

GWAS was performed a second time using BayesB for all traits, where the resulting Manhattan plots are depicted in Figure 2. This method tested the association between the dEBVs of traits with genomic regions set as windows instead of individual SNPs. The number of significant windows was 20, 34, 24, 26, 24, 20, 24, 19, 21, 21, and 31 for ADG, BF, DFI, FCR, RFI, RG, RIG, KR, LD, NVD, and TPD, respectively. Many of these windows were located on SSC 1, 7, 8, 9, and 10. Significant SNPs from LMM that fall within the significant windows from BayesB were defined as overlapping significant SNPs hereafter in this article. Only BF, DFI, FCR, LD, NVD, and TPD had overlapping SNPs that were significant from both methods. These SNPs are summarized in Table 3 as well as their minor allele frequencies (MAF) and percent contribution to the overall phenotype. Most SNPs contributed less than 1%; however, rs336943540 and rs330992687 on SSC 7, and rs338030731 and rs323028631 on SSC 10 contributed 1.66%, 1.71%, 1.30%, and 1.30%, respectively, to FCR, while rs336943540 and rs330992687 contributed 1.66% and 1.20% to BF. Therefore, the total percent contribution from the SNPs identified by both LMM and BayesB is 14.23% for FCR and 8.29% for BF.

**Table 3:** Overlapping significant SNPs obtained from the linear mixed model and Bayesian B model.

Trait	SSC	Start (bp)	End (bp)	SNP	MAF	Var. Exp	*P*-value*
**FCR**	7	30502880	30993927	rs336943540	0.31	1.66	2.36E-13
				rs81280321	0.49	0.61	6.87E-05
				rs81280322	0.49	0.59	8.29E-05
				rs337714191	0.19	0.50	5.83E-05
				rs332708122	0.49	0.60	7.49E-05
				rs80909406	0.19	0.51	5.90E-05
				rs323761835	0.46	0.78	2.02E-05
				rs345292085	0.47	0.83	1.16E-05
				rs331539554	0.47	0.75	2.80E-05
				rs80918904	0.47	0.81	1.41E-05
				rs330992687	0.33	1.71	2.13E-11
	10	26528469	26884027	rs696726562	0.47	0.92	2.35E-10
				rs338030731	0.40	1.30	4.27E-13
				rs322374397	0.49	0.72	5.06E-08
				rs323028631	0.40	1.30	4.33E-13
	13	23019809	23426699	rs320459013	0.15	0.65	9.75E-08
**DFI**	10	26528469	26884027	rs334442897	0.40	0.20	1.43E-10
				rs696726562	0.47	0.13	6.28E-08
				rs338030731	0.40	0.20	1.79E-10
				rs322374397	0.49	0.12	8.93E-07
				rs323028631	0.40	0.20	2.00E-10
**BF**	7	30502880	30993927	rs336943540	0.31	1.66	3.74E-11
				rs81280321	0.49	0.97	1.42E-05
				rs81280322	0.49	0.94	1.99E-05
				rs337714191	0.19	0.67	4.98E-05
				rs332708122	0.49	0.97	1.41E-05
				rs330992687	0.33	1.20	3.42E-06
	7	97584287	97954258	rs330032123	0.47	0.33	8.96E-20
				rs326953479	0.47	0.34	1.46E-20
	10	26528469	26884027	rs334442897	0.40	0.27	7.35E-14
				rs696726562	0.47	0.14	1.08E-08
				rs338030731	0.40	0.26	9.52E-14
				rs323028631	0.40	0.26	1.46E-13
				rs81345562	0.16	0.09	1.00E-06
				rs333627827	0.09	0.07	3.00E-06
	12	25024143	25354008	rs327303574	0.38	0.12	1.96E-08
**LD**	7	97584287	97954258	rs330032123	0.47	0.45	2.27E-07
				rs326953479	0.47	0.45	2.46E-07
**TPD**	14	132566098	132980840	rs80916286	0.17	0.38	4.22E-05
				rs345812860	0.17	0.40	2.26E-05
**NVD**	2	21781	469146	rs324063164	0.31	0.34	6.40E-05
				rs330530611	0.31	0.33	8.73E-05
				rs320993727	0.31	0.33	7.30E-05
				rs324933303	0.31	0.35	4.22E-05

* Obtained from linear mixed model (LMM)

Abbreviations: FCR, feed conversion ratio; DFI, daily feed intake; BF, backfat thickness; LD, loin depth; SSC, Sus scrofa chromosome; bp, base pair; MAE, minor allele frequency; Var. Exp, percentage of additive variance explained by each SNP.

### Candidate genes and functional enrichments

Using the significant SNPs from LMM, genes were annotated through the biomaRT package (Durinck et al. 2009) and used to identify biological pathways involved in the studied traits. The number of identified named genes were 54, 196, 129, 120, 88, 79, 100, 0, 44, 56, and 21 for ADG, BF, DFI, FCR, RFI, RG, RIG, KR, LD, NVD, and TPD, respectively. The most significant SNPs and nearest candidate genes for each trait are summarized in Table 4. The overlap of significant SNPs across several traits led to the identification of *PACSIN1, PTCH1, ANKS1A, ADIPOR1*, and *ITPR3* as candidate genes influencing multiple traits (BF, DFI, FCR, RFI, RG, RIG). Other important candidate genes to note associated with ADG, BF, FCR, and DFI included *ABCD4*, *MC4R*, and *CDH20.* The annotated candidate genes were uploaded to PANTHER to identify associated biological pathways. The most reoccurring pathways across traits were the gonadotropin-releasing hormone receptor (GnRH) and the platelet-derived growth factor (PDGF) signaling pathways (Table 5).

**Table 4:** Most significant SNPs obtained from linear mixed model (LMM) and nearest gene for each the studied trait.

Trait	SSC	Most significant SNP	Position bp	*P*-value	Nearest gene
**RFI**	7	rs336943540	30502880	3.30E-09	*PACSIN1*
	10	rs81422613	26091506	1.88E-09	*ERCC6L2*
**FCR**	7	rs336943540	30502880	2.36E-13	*PACSIN1*
	10	rs334442897	26699078	3.55E-13	*PTCH1*
**ADG**	1	rs81284646	161824864	6.12E-09	*SEC11C*
		rs81349297	161853405	4.64E-09	*ZNF532*
**DFI**	10	rs334442897	26699078	1.43E-10	*FANCC*
		rs338030731	26701116	1.79E-10	*MIR27B*
**BF**	7	rs330032123	97584287	8.96E-20	*VSX2*
		rs326953479	97618073	1.46E-20	*ABCD4*
**LD**	7	rs330032123	97584287	2.27E-07	*ABCD4*
		rs326953479	97618073	2.46E-07	*SYNDIG1L*
**KR**	–	–	–	–	*-*
**RG**	10	rs81231356	24927504	8.31E-11	*CYB5R1*
		rs338030731	26702300	1.09E-10	*PTCH1*
**RIG**	7	rs336943540	30502880	3.30E-09	*PACSIN1*
	10	rs81422613	26091506	1.89E-09	*ERCC6L2*
**TPD**	6	rs321966293	29780931	7.68E-06	*CES5A*
		rs339247109	30303413	6.15E-06	*IRX6*
**NVD**	2	rs324933303	159141	4.22E-05	*IFITM5*
	4	rs80856655	105312765	3.31E-05	*NGF*

*Note:* RFI, residual feed intake; FCR, feed conversion ratio; ADG, average daily gain; DFI, daily feed intake; BF, backfat thickness; LD, loin depth; KR, Kleiber’s ratio; RG, residual gain; RIG, residual intake and gain; TPD, total time spent eating per day; NVD, number of visits to the feeder per day; SSC, Sus scrofa Chromosome; bp, base pair.

**Table 5:** Top pathway associated with trait from PANTHER analysis.

Trait	N Genes	Genes	Most prominent pathway
**RFI**	5	*ACVR2B, ADIPOR1, ITPR3, NOS1, PRKAB1*	Gonadotropin-releasing hormone receptor pathway
**FCR**	5	*ACVR2B, ADIPOR1, ITPR3, MAPK13, MAPK14*	Gonadotropin-releasing hormone receptor pathway
**ADG**	2	*SERPINB2, BCL2*	CCKR signaling pathway
**DFI**	6	*ADIPOR1, SMAD3, MAPK13, ITPR3, ACVR2B, MAPK14*	Gonadotropin-releasing hormone receptor pathway
**BF**	4	*RPS6KL1, ELF3, ITPR3* *SPDEF*	PDGF signaling pathway
**LD**	6	*NOS1, ADIPOR1, ITPR3, ACVR2B, PRKAB1, ACVR2B*	Cobalamin biosynthesis
**RG**	3	*ITPR3, ELF3, SPDEF*	PDGF signaling pathway
**RIG**	3	*ACVR2B, ADIPOR1, ITPR3*	Gonadotropin-releasing hormone receptor pathway
**NVD**	2	*BMP10, HRAS*	TGF-β signaling pathway

*Note*. Traits with only one gene or fewer identified to be involved in a pathway was omitted (Kleiber’s ratio and Total time spent eating per day). RFI, residual feed intake; FCR, feed conversion ratio; ADG, average daily gain; DFI, daily feed intake; BF, backfat thickness; LD, loin depth; RG, residual gain; RIG, residual intake and gain; NVD, number of visits to the feeder per day; CCKR, cholecystokinin; PDGF, platelet-derived growth factor; TGF-β, transforming growth factor-β.

## Discussion

Growth and FE are among the most important traits in the selection of Canadian pigs due to their direct impact on the economic profitability of the industry by reducing feeding costs (Patience et al. 2015). Identifying genetic markers associated with these traits provides valuable insights into their underlying genetic mechanisms. Therefore, numerous studies used GWAS to identify SNPs associated with FE measures such as FCR and RFI in pigs (Do et al. 2014). Likewise, feeding behavior as one of the biggest factors contributing to FE and production traits that are also highly valued were extensively studied using GWAS analyses (Silva et al. 2019; Ding et al. 2018). With increased application of GWAS in the field of animal breeding, various methods are being continuously developed to support diverse study designs to investigate the genetic architecture underlying traits of interests. For example, multi-trait GWAS can simultaneously analyze multiple traits, offering increased statistical power and identification of pleiotropic loci (Galesloot et al. 2014). Likewise, methods such as single-step GWAS, which combines genomically derived relationships (G) with population-based pedigree relationships (A) into a H matrix, allowed for improved integration of all available data and reduction of noise (Hong et al. 2025). Additionally, single-step GWAS was successful in the identification of candidate genes for maternal genetic effects in traits such as birth and weaning weight in sheep (Khazaei-Koohpar et al. 2024). As maternal genetic effects can contribute substantially to traits such as fattening performance, post-weaning growth, and intramuscular fat content, performing GWAS for maternal genetic effects in addition to direct additive genetic effects is increasing becoming a point of interest (El Nagar et al. 2023). In the case of this study, a simple single-trait LMM was selected, as multi-trait GWAS and single-step GWAS can be computationally challenging and to allow for independent interpretation of each trait. Combined with BayesB, marker‑based Bayesian variable selection and mixed‑model association testing was achieved for 11 traits related to FE, production, and feeding behavior. This investigation led to the identification of significant markers for all traits except for KR. The absence of significant SNPs for KR is likely attributable to the trait’s low to moderate heritability, indicating that environmental factors may contribute more to its variability than genetic factors (Rezende et al. 2022).

LMM is one of the most widely used methods in GWAS and is known for its effectiveness in detecting associations between genetic variants and phenotypic traits (Wang et al. 2016). However, issues such as genomic background blurring true signals, false positive markers with high linkage disequilibrium to a causal gene as well as overly stringent thresholds (Bonferroni) can hinder its ability to detect true associations. As a result, alternative approaches such as Bayesian methods are also explored. In this study, both LMM and BayesB were applied, and only six traits (BF, DFI, FCR, LD, TPD, and NVD) showed overlapping significant SNPs or genomic windows. Although differences in results may stem from fundamental variations in model assumptions or statistical thresholds, they may also reflect trait-specific genetic architectures. Since BayesB assumes that most loci have no genetic variance and only a few have measurable effects, it may fail to detect quantitative trait loci (QTL) with smaller effects (Meuwissen et al. 2001; Shi et al. 2021). Therefore, it is better suited for traits that are influenced by a few genes with large effects. Traits that lacked overlapping significant SNPs from the LMM and BayesB models may have polygenic tendencies, controlled by many genes that each contribute a small proportion of the variance, leading to the discrepancies in results.

Many traits (BF, DFI, FCR, RFI, RG, and RIG) had shared significant SNPs on SSC 7, particularly around 30.3–30.9 Mb, 33.1–33.8 Mb, and 34.4–34.5 Mb, and on SSC 10 at 24.9 Mb and 26.0–26.7 Mb. This suggests that these traits may be regulated by similar genetic mechanisms. While previous studies have reported significant SNPs on SSC 7 and SSC 10 for FCR and RFI, the specific regions identified in this study have yet to be mentioned in literature (Onteru et al. 2013; Do et al. 2014; Bai et al. 2017), indicating that these SNPs may be novel findings. Interestingly, nearby regions on SSC 7 around  34.0 Mb and 36.0 Mb have been associated with and confirmed to hold QTLs for FCR in White Duroc × Erhualian F2 intercrossed pigs (Guo et al. 2015). The regions 34.4-34.5 Mb also held significance SNPs in the present study, albeit to a lesser extent than the 30.5 Mb region, suggesting that these loci may be evolutionarily conserved and that FE traits likely have a shared genetic basis across breeds (Guo et al. 2015). The close physical proximity between the 30.5 Mb region and the nearby regions at 34.0 Mb and 36.0 Mb on SSC 7 raises the possibility that the SNPs within this range are in linkage disequilibrium and located near a true causal variant. Additionally, the 34.0 Mb region on SSC 7 was previously found to explain 7.63% of the phenotypic variance of FCR while also signficantly affecting ADG (Zhang et al. 2009). This further supports the idea that SSC 7 harbors multiple evolutionarily conserved QTLs possibly within the 30.5–34.0 Mb interval, contributing not only to FCR but also to other FE and production traits. Though pleiotropic QTLs between FCR and other FE traits such as RFI and DFI have previously been identified on SSC 1 and SSC 7 in Duroc pigs, additional analyses are required to confirm such tendencies in the above-identified regions (Ding et al. 2018). Overall, further research in the SSC 7 region from 30.5 to 34.0 Mb, such as fine mapping or the characterization of the linkage disequilibrium patterns is highly recommended to better understand the QTLs influencing FE and production traits.

Various genes were annotated within a 0.5 Mb window upstream and downstream of the significant SNPs mentioned above. Given the overlapping significant SNPs across multiple traits, several genes, including *PACSIN1, PTCH1, ADIPOR1*, and *ITPR3*, were repeatedly identified. Notably, many of these genes are involved in regulating cell growth and tumor suppression. *PACSIN1* (Protein kinase C and casein kinase substrate in neurons 1) plays a role in neuromorphogenesis, receptor trafficking, and synaptic plasticity (Dumont and Lehtonen 2022). It has been identified as a prominent candidate gene, previously associated with LD and loin muscle area in Duroc pigs (Zhuang et al. 2019), as well as carcass length, foot weight, head weight, slaughter body weight, and BF in Large White×Minzhu intercross pigs (Liu et al. 2014). These findings highlight the strong potential of *PACSIN1* for the selection of multiple economically important traits, particularly production traits, while also suggesting relevance for FE. *PTCH1* (Patched 1) regulates cell growth, development, and the suppression of tumors. In mice, *PTCH1* is also involved in cholesterol transport and overall cholesterol metabolism (Li et al. 2019). Further studies are required in pigs on how *PTCH1* is involved in metabolism and to confirm its role in FE or production traits. *ADIPOR1* (Adiponectin Receptor 1) is a gene that codes for adiponectin, which is a hormone produced by adipocytes and regulates glucose and lipid metabolism in mice (Zhao et al. 2021). *ADIPOR2*, another adiponectin receptor abundantly expressed in the uterus and ovaries, is associated with productivity traits such as total number of piglets born and litter weight at weaning in Duroc, Landrace, and Yorkshire pigs (Jafarikia et al. 2015). Adiponectin concentrations in serum are negatively associated with visceral fat accumulation, plasma glucose levels, insulin levels, and body mass index in humans; however, further studies are required in pigs to determine its function outside of reproductive traits (Brochu-Gaudreau et al. 2010). *ITPR3* encodes type 3 inositol-tri-phosphate receptor (IP3R3), which is involved in the regulation of Ca^2+^ release from the endoplasmic reticulum (Cabello-Murgui et al. 2024). Its overexpression has been associated with cancer and Charcot Marie Tooth disease, a sensory-motor neuropathy, in humans. In mice, under-expression or mutations in *ITPR3* can impair taste perception, particularly for sweet, bitter, and umami flavors (Tordoff and Ellis 2013). This effect on taste perception can be a potential factor affecting feed intake and feeding behavior. However, further studies must be conducted on how this gene contributes to FE, production, and feeding behavior traits in pigs.

In addition to the above-mentioned novel candidate genes, this study identified several candidate genes that are well known in the literature. ATP-binding cassette subfamily D member 4 (*ABCD4*), located on SSC 7 between 97.5 Mb and 97.6 Mb, was the gene nearest the most significant SNP for BF and LD. Although a direct connection between *ABCD4* with production traits such as BF and LD in pigs has yet to be clearly identified, it has been noted as a top candidate gene involved in the variation of number of rib pairs in Beijing black pigs (Niu et al. 2022). *ABCD4* being a member of the ATP-binding cassette transporter superfamily is involved with the transport of the vitamin B12, which is an important component of the Wnt pathway that is involved in the development of the ribs and vertebrae (Ciappio et al. 2011). Given that BF and LD share close anatomical proximity to the ribs and the vertebrae, this overlap in this candidate gene may suggest *ABDC4* relates to multiple phenotypes that are developmentally linked to the same thoracic region. Additionally, *ABCD4* is a prominent candidate gene in commercial crossbred pigs such as Duroc × (Landrace × Yorkshire) for body conformation traits such as body length, body height, chest, abdominal, and waist circumference (Deng et al. 2023; Mozduri et al. 2025). These findings support *ABCD4* as a strong potential candidate gene for various traits that are within the thoracic region. Other noteworthy candidate genes that are associated with ADG, BF, FCR, and DFI are melanocortin 4 receptor (*MC4R*) and cadherin 20 (*CDH20*) both located on SSC 1. Except for ADG, significant SNPs on SSC 1 are less emphasized in the results compared to SSC 7 and 10; however, *MC4R* and *CDH20*, along with *LEPR* have been noted as strong candidate genes in literature for growth and fatness. More specifically, such genes are involved in fat deposition, metabolism, and energy balance (Mozduri et al. 2025). Therefore, in commercial Canadian pigs, all three genes have been identified to be associated with various primal cut traits such as BF, LD, belly fat, total fat, ham fat, picnic fat, and butt fat (Mozduri et al. 2025). *MC4R* and genes within the cadherin family (*CDH19* and *CDH7*) are famous candidate genes for ADG and DFI in both purebred populations such as Yorkshires and crossbreeds between Landrace and Large White (Onteru et al. 2013, Silva et al. 2019). As FCR is derived from using both ADG and DFI, it is of no surprise that *MC4R* and *CDH20* are shared as candidate genes. Overall, the identified candidate genes from this study are in concordance with those of the literature, validating the link between growth and fat-based genes with FE and production traits.

The GO analysis revealed several biological pathways enriched among the candidate genes, including the GnRH signaling pathway, the PDGF signaling pathway, and the transforming growth factor-β (TGF-β) signaling pathway. Of these pathways, the GnRH signaling pathway has been previously associated with FE in pigs (Xu et al. 2021) as well as other species such as cattle, sheep, and mink (Kiyma et al. 2000; Taussat et al. 2020; Davoudi et al. 2025). GnRH signaling pathways regulate the secretion of gonadotropic hormones, namely luteinizing hormone and follicle-stimulating hormone (FSH; Adams 2005). Interestingly, recent studies suggest that FSH may also regulate fat accumulation in pigs and humans, where individuals that secrete higher levels of FSH tend to accumulate more fat (Han et al. 2021; Chen et al. 2023). These findings support the idea that GnRH signaling plays a significant role in FE and growth, not only in pigs but across multiple species. However, additional studies are needed to further explore the connection between FE and reproductive function. The PDGF signaling pathway is crucial in the growth and spread of cancer but also regulates the metabolism of cyclic adenosine monophosphate (cAMP), a molecule required for cell signaling (Graves et al. 1996). cAMP plays a direct role in metabolic regulation, including glycogen metabolism and the olfactory transduction pathway (Ferguson and Zhao 2016). Notably, the olfactory transduction pathway has previously been associated with RFI in Duroc pigs (Do et al. 2014), linking the results in this study to existing literature. The TGF-β signaling pathway, while also associated with cancer, is essential for embryonic development, wound healing, tissue repair, and immune homeostasis (Deng et al. 2024). Elevated levels of TGF-β are associated with obesity in humans and mice, as it causes a long protein cascade leading to adipocyte lipogenesis and gluconeogenesis (John et al. 2025). Based on these pathways, it is notable that some genes like *ACVR2B, ITPR3*, and *MAPK13* are involved in multiple pathways, strengthening their candidacy as key genes regulating FE, production, and feeding behavior in pigs. Overall, additional research is necessary to understand how these biological pathways influence economically important traits in pigs, especially since much of the current functional knowledge is derived from studies in humans and mice.

## Conclusion

In conclusion, GWAS analysis using both methods of LMM and BayseB identified a broad range of statistically significant SNPs, with the number of significant markers ranging from 6 to 101 SNPs across different traits, with 24 shared in BF, DFI, FCR, RFI, RG, and RIG on SSC 7 and 10. Similarly, the number of annotated genes surrounding these SNPs varied by trait, ranging from 21 to 196, with 71 shared between the above-mentioned six traits. Several genes, including *PACSIN1, PTCH1, ADIPOR1*, and *ITPR3*, were found to have known roles in metabolism and may also contribute to FE, production and feeding behavior traits in pigs. Previously identified genes such as *MC4R* and *CDH20* have also been found to be associated with ADG, BF, FCR, and DFI in this study. Enrichment analysis further revealed that many of the candidate genes were involved in key biological pathways, such as the GnRH signaling, the PDGF signaling, and the TGF-β signaling pathways. Overall, this study identified 24 SNPs associated with six economically important traits in Canadian Duroc pigs. These findings have potential applications in genomic selection programs, highlighting the putative contribution of these genes to FE, production, and feeding behavior traits and their relevance for improving productivity in pig populations.

## Supplementary Material

skag148_Supplementary_Data

## Abbreviations

ADG: average daily gain adjusted to 120 kg of body weight
BW: body weight
BF: backfat thickness adjusted to 120kg of body weight
CCSI: Canadian Center for Swine Improvement
DFI: daily feed intake
FCR: feed conversion ratio
FE: feed efficiency
GWAS: genome-wide association studies
KR: Kleiber ratio
LD: loin depth adjusted for 120 kg of body weight
LMM: linear mixed model
NVD: number of visits to the feeder per day
PIC: pig improvement company
QTL: quantitative trait loci
RF: random forest
RFI: residual feed intake
RG: residual gain
RIG: residual intake and gain
SNP: single nucleotide polymorphism
TPD: total time spent eating per day
