# Supplementary material for: Genome-wide association studies for feed efficiency, production and feeding behavior traits in Canadian purebred Duroc pigs
Source: J Anim Sci. 2026 May 9;104:skag148. doi: 10.1093/jas/skag148 (PMC13215767; doi:10.1093/jas/skag148)
Supplement: skag148_Supplementary_Data [file skag148_supplementary_data.docx]

**Supplementary**


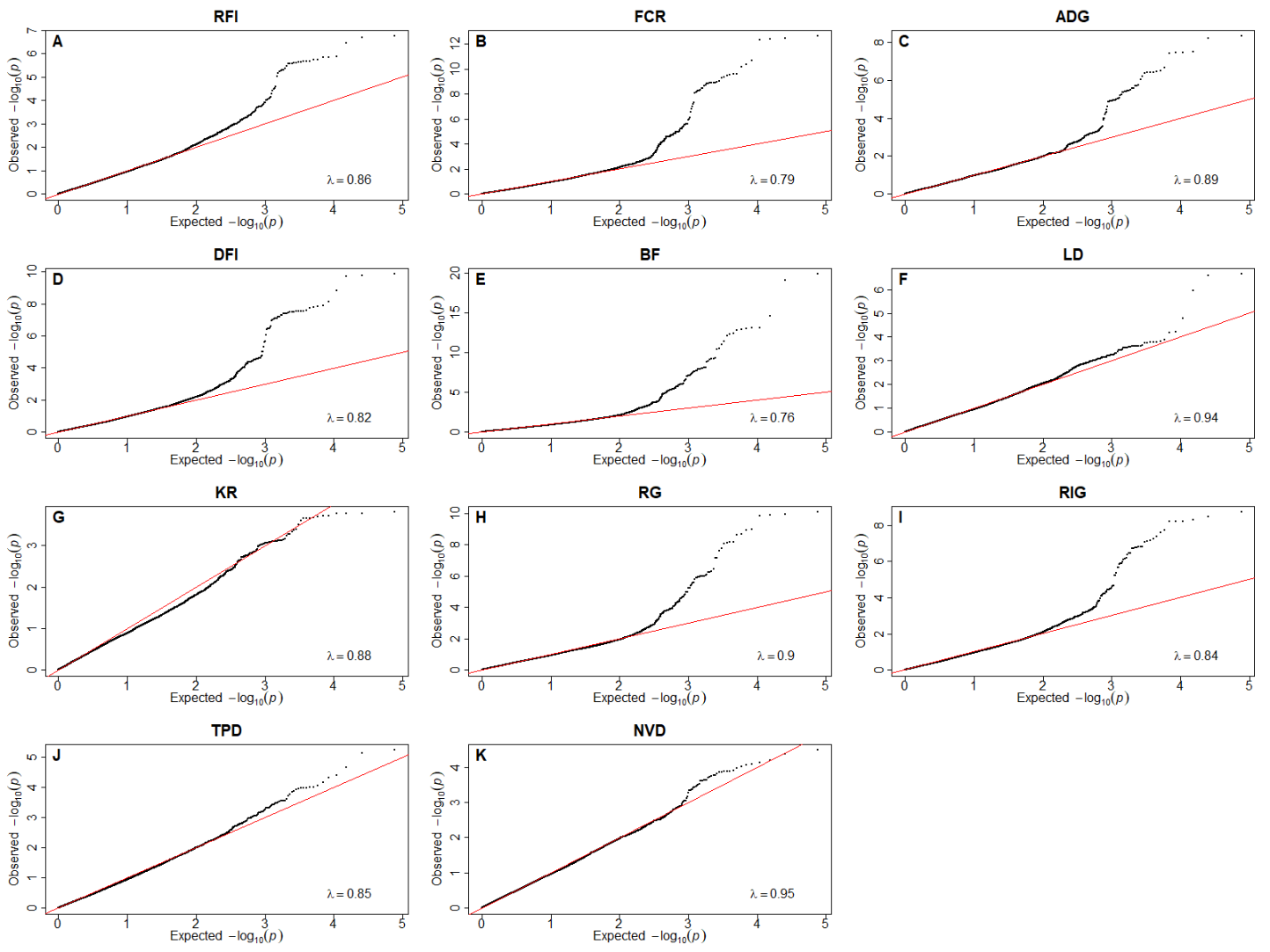


*Supplementary Figure 1. QQ plots of the observed and expected log10 of the p-value for each trait resulting from Linear Mixed Model (LMM). (a) Residual feed intake; (b) Feed conversion ratio; (c) Average daily gain; (d) Daily feed intake; (e) Backfat thickness; (f) Loin depth; (g) Kleiber’s Ratio; (h) Residual Gain; (I) Residual intake and gain; (j) Total time spent eating per day; (k) Number of visits to the feeder per day.*


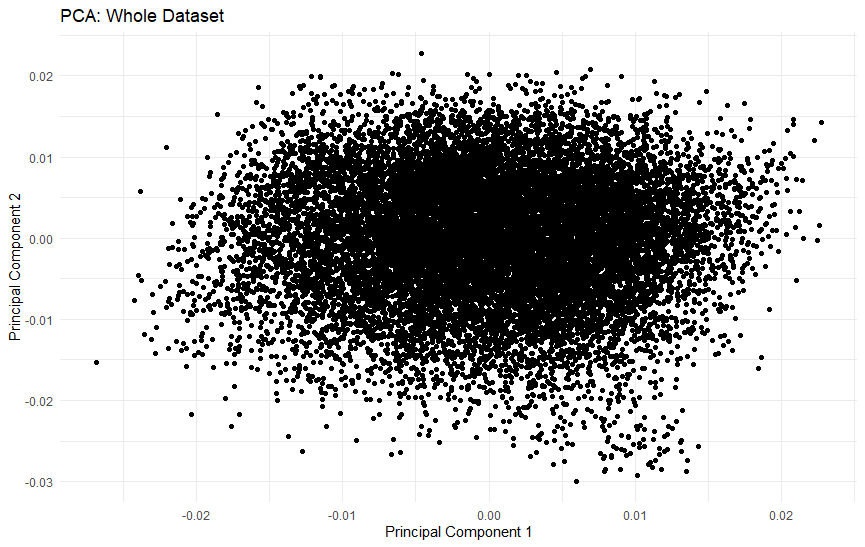


*Supplementary Figure 2: Principal Component Analysis*


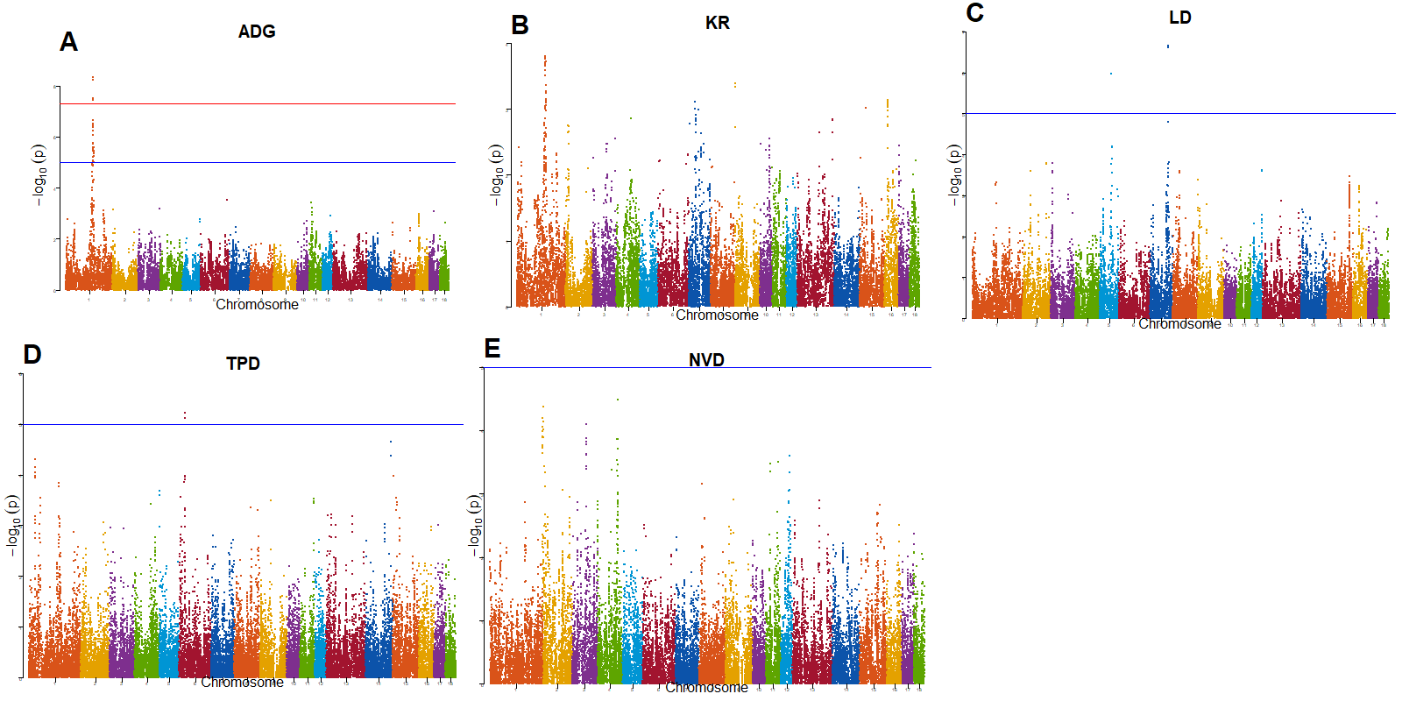


*Supplementary Figure 3: Manhattan plots of the (a) Average daily gain (ADG), (b) Kleiber’s ratio, (c) Loin depth, (d) Total time spent eating per day, and (e) Number of visits to the feeder per day resulting from Linear Mixed Model (LMM).*


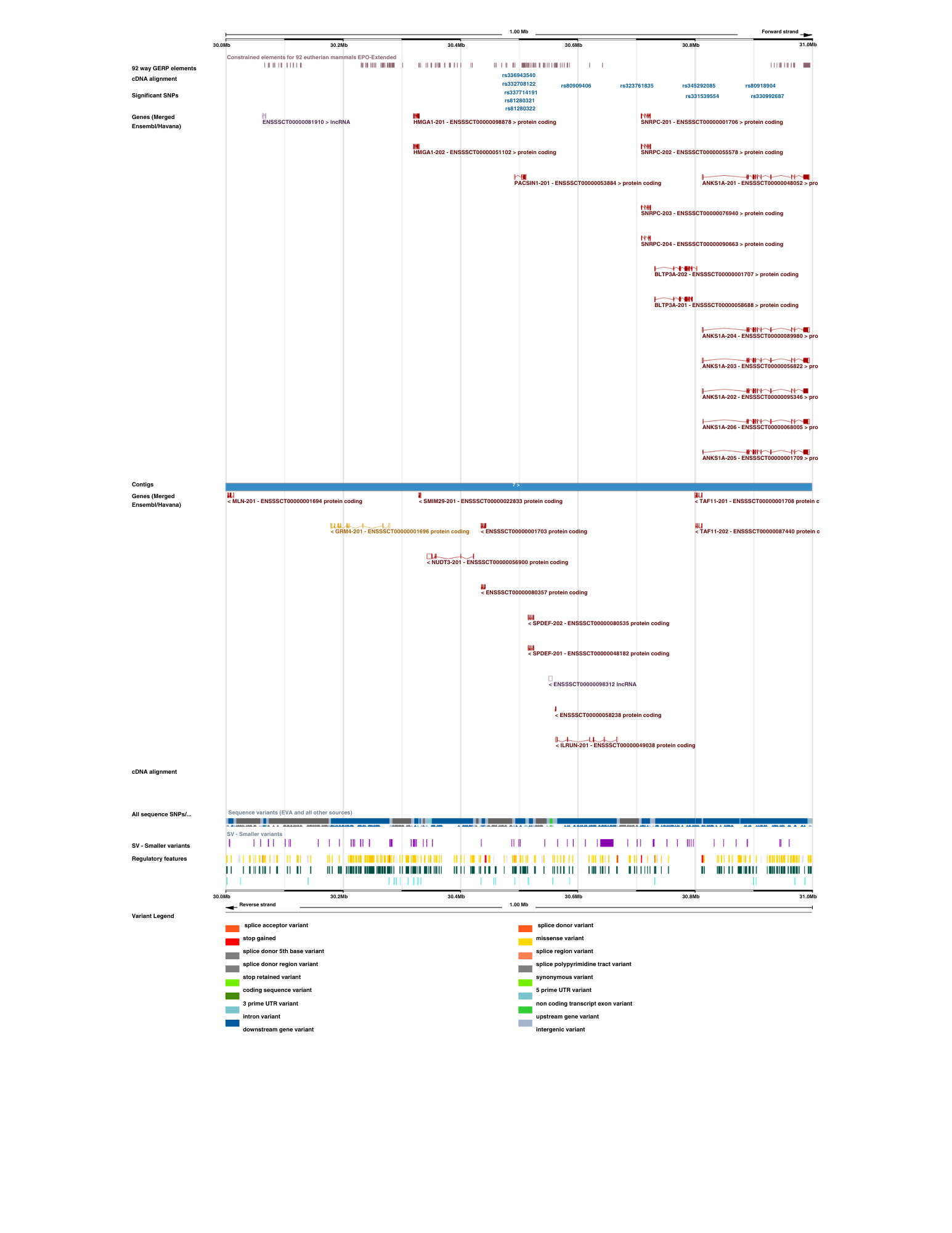


*Supplementary Figure 4: Significant SNP locations and gene annotations of SSC 7 30.0–31.0 Mb genomic region based on Ensembl (Sscrofa 11.1).*


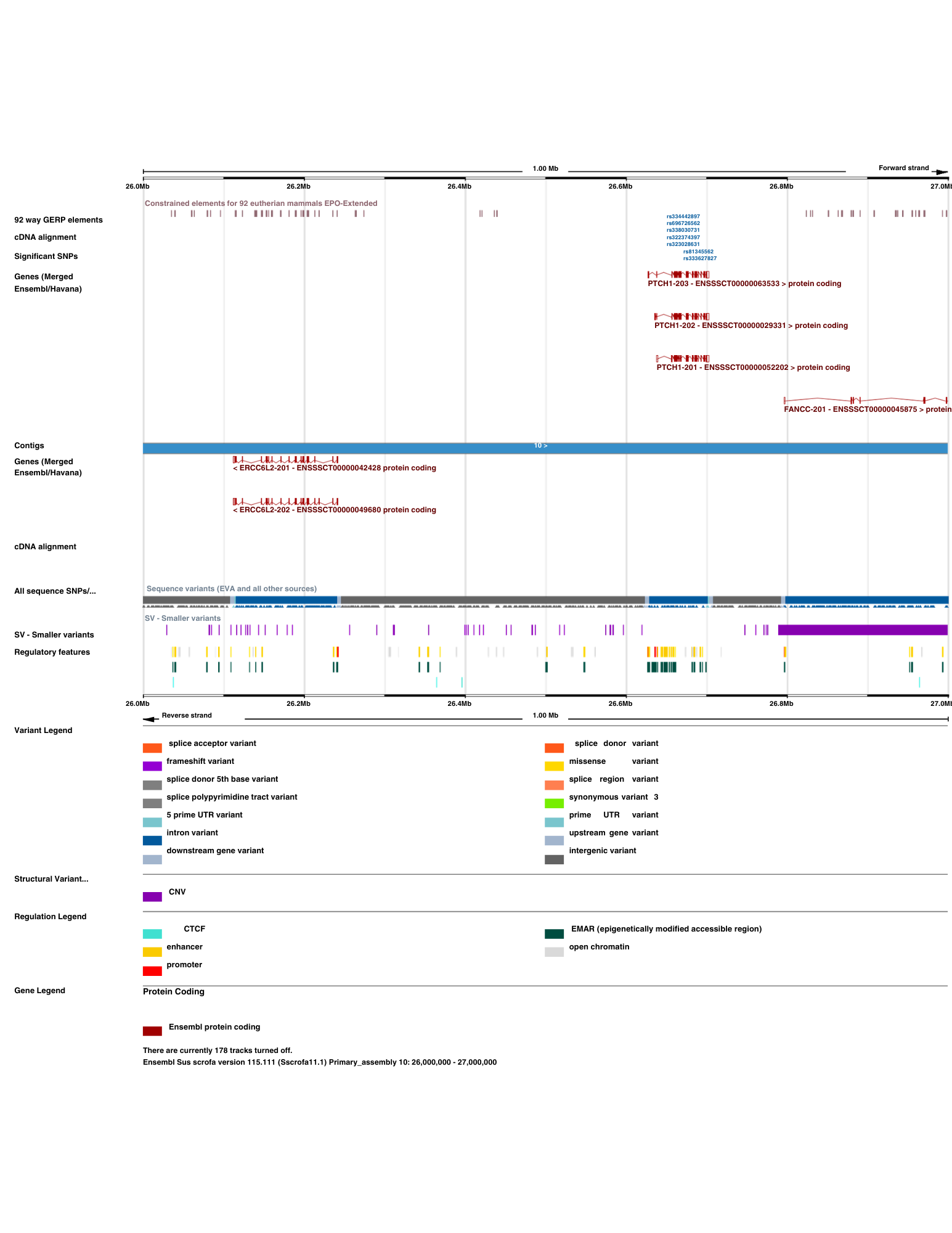


*Supplementary Figure 5: Significant SNP locations and gene annotations of SSC 10 26.0–27.0 Mb genomic region based on Ensembl (Sscrofa 11.1).*
